# Parameters for Fabricating Nano-Au Colloids through the Electric Spark Discharge Method with Micro-Electrical Discharge Machining

**DOI:** 10.3390/nano7060133

**Published:** 2017-06-02

**Authors:** Kuo-Hsiung Tseng, Meng-Yun Chung, Chaur-Yang Chang

**Affiliations:** Department of Electrical Engineering, National Taipei University of Technology, Taipei 10608, Taiwan; avicwizard@hotmail.com (M.-Y.C.); cychang@ntut.edu.tw (C.-Y.C.)

**Keywords:** electric spark discharge method, micro-electrical discharge machining, nano-Au colloid, nanosuspension stability

## Abstract

In this study, the Electric Spark Discharge Method (ESDM) was employed with micro-electrical discharge machining (m-EDM) to create an electric arc that melted two electrodes in deionized water (DW) and fabricated nano-Au colloids through pulse discharges with a controlled on–off duration (T_ON_–T_OFF_) and a total fabrication time of 1 min. A total of six on–off settings were tested under normal experimental conditions and without the addition of any chemical substances. Ultraviolet–visible spectroscopy (UV–Vis), Zetasizer Nano measurements, and scanning electron microscopy–energy dispersive X-ray (SEM–EDX) analyses suggested that the nano-Au colloid fabricated at 10–10 µs (10 µs on, 10 µs off) had higher concentration and suspension stability than products made at other T_ON_–T_OFF_ settings. The surface plasmon resonance (SPR) of the colloid was 549 nm on the first day of fabrication and stabilized at 532 nm on the third day. As the T_ON_–T_OFF_ period increased, the absorbance (i.e., concentration) of all nano-Au colloids decreased. Absorbance was highest at 10–10 µs. The SPR peaks stabilized at 532 nm across all T_ON_–T_OFF_ periods. The Zeta potential at 10–10 µs was −36.6 mV, indicating that no nano-Au agglomeration occurred and that the particles had high suspension stability.

## 1. Introduction

Across the world and throughout history, gold has been regarded as a symbol of nobility. Its glittering beauty attracts the eye. Gold is a highly stable element used for decoration and added as an antioxidant to tea, wine, and certain foods [1]. However, at the nanoscale, gold turns dark because it absorbs the light of certain wavelengths [2]. In addition to being medically exploited for its antioxidant effects, nano-Au has a great potential for use in DNA chips designed to detect hereditary diseases; thus, its fabrication has been extensively studied [3].

Currently, the preparation methods for nanometal colloids are primarily divided into physical and chemical methods. However, the typical chemical methods require adding some chemical agents, so the products contain other derivatives [4]. Moreover, nanometal colloids prepared through chemical methods are easily contaminated during preparation. Human beings can be harmed if they are exposed to nanoscale particles that cause ailments of the lungs and other organs [5,6]. Therefore, to solve the problem of dust drift in the processing environment is a major challenge of nanotechnology research.

Several studies have discussed the application of Electrical Discharge Machining (EDM) in nanomanufacturing [7,8,9]. The experimental conditions for implementing EDM in nanomanufacturing are described as follows: (1) one electrode is attached to the bottom of a vessel with a direct current (DC) power source, and deionized water (DW) is subsequently added to the vessel [10]; (2) another electrode is attached to a servomechanism, which adjusts the gap between the two electrodes; (3) the on–off duration of pulse discharge (T_ON_–T_OFF_), current intensity, and other parameters are specified on a control panel [11]; and (4) a magnetic stirrer is used to rotate a magnet in the vessel, thereby stirring the DW in the vessel and distributing nanoparticles evenly in the liquid. Nano-Au colloids prepared through EDM have exhibited absorbance spectra with surface plasmon resonance (SPR) peaks at 544 nm and a zeta potential at −40 mV, as observed through ultraviolet–visible spectroscopy (UV–Vis) and Zetasizer Nano measurement. However, fabricating nano-Au colloids through EDM entails considerable costs, and the fabrication process cannot be monitored in real time. Furthermore, wear on the machine’s components due to long-term use undermines the efficiency of the process. The efficiency of fabricating nanometal colloids is proportional to the discharge success rate. Observing the discharge success rate in real time during fabrication can help to resolve malfunctions and to improve the quality of the products. For example, when the two electrodes become misaligned, the success rate declines, but that success rate can be regained by realigning the electrodes in a straight line. Traditional EDM can monitor only the current of the gap between the electrodes; it cannot identify the discharge success rate. To solve this problem, the discharge success rate circuitry in this study was designed to monitor the discharge success rate in real time. To ameliorate the shortcomings of EDM [12], this study proposed a micro-Electrical Discharge Machining (m-EDM) for fabricating nano-Au; this m-EDM was found to be suitable for nanomanufacturing.

## 2. Results

### 2.1. Nano-Au Colloid Preparation

Optimal Parameters for Fabricating Nano-Au Colloids through m-EDM

Table 1 lists the parameters used for fabricating nano-Au colloids through m-EDM. At the standard temperature and pressure, a proportional–integral–derivative (PID) controller was configured with the proportional controller setting Kp = 0.21, integral controller setting Ki = 0.25, and derivative controller setting Kd = 0.015; 99.99% pure metal electrodes with a diameter of 1 mm were prepared in 100 mL of DW at different periods of T_ON_–T_OFF_ (for a total discharge time of 1 min per configuration) to produce nano-Au colloids. A necessary condition of m-EDM with high process efficiency is that the electrode gap in the discharge process must be maintained within a small range. To meet the conditions, proportional–integral–derivative (PID) control was used. Through PID control, the positive and negative rotation of the motor can be regulated to maintain the electrodes within a range of 30 µm.

**Figure 1:**
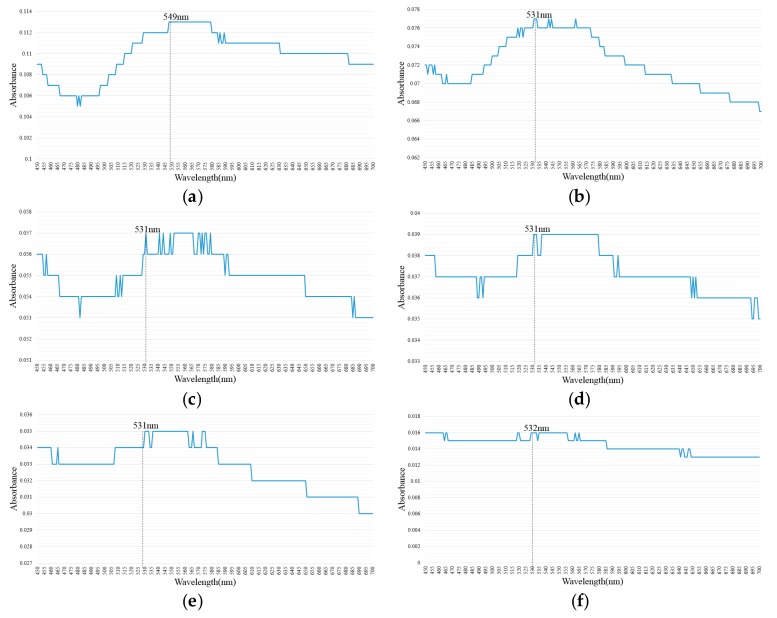
Daily ultraviolet–visible spectroscopy (UV–Vis) analysis results of nano-Au colloids fabricated at T_ON_–T_OFF_ values of (**a**) 10–10 µs; (**b**) 20–20 µs; (**c**) 30–30 µs; (**d**) 40–40 µs; (**e**) 50–50 µs; (**f**) 100—100 µs.

On the basis of daily UV–Vis analysis results (Figure 1), nano-Au colloids prepared with T_ON_–T_OFF_ values of 10–10, 20–20, 30–30, 40–40, 50–50, and 100–100 µs had SPR peaks of 549, 531, 531, 531, 531, and 532 nm, respectively, with corresponding absorbance values of 0.113, 0.077, 0.057, 0.039, 0.035, and 0.016. The nano-Au colloid prepared at a T_ON_–T_OFF_ of 10–10 µs had the highest absorbance and concentration, whereas that prepared at a T_ON_–T_OFF_ of 100–100 µs had the lowest absorbance and concentration. The SPR peak was slightly higher at 10–10 µs (549 mm) than at other T_ON_–T_OFF_ periods.

**Figure 2:**
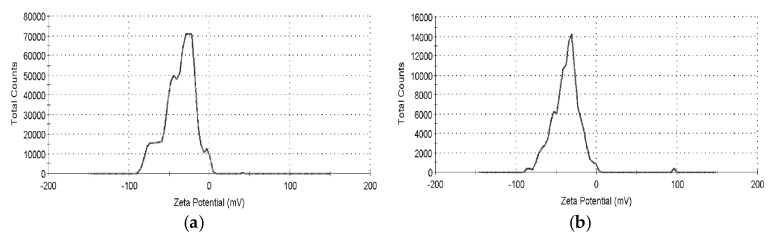
Daily Zeta potential analysis results of nano-Au colloids fabricated at T_ON_–T_OFF_ settings of (**a**) 10–10 µs; (**b**) 50–50 µs.

The Zeta potential was −36.6 mV for the nano-Au colloid prepared at 10–10 µs (Figure 2a) and −37.6 mV for that prepared at 50–50 µs (Figure 2b). An absolute Zeta potential value of over 30 mV indicates high suspension stability in the nanoparticles. Therefore, nano-Au colloids fabricated through m-EDM exhibited high suspension stability.

**Figure 3:**
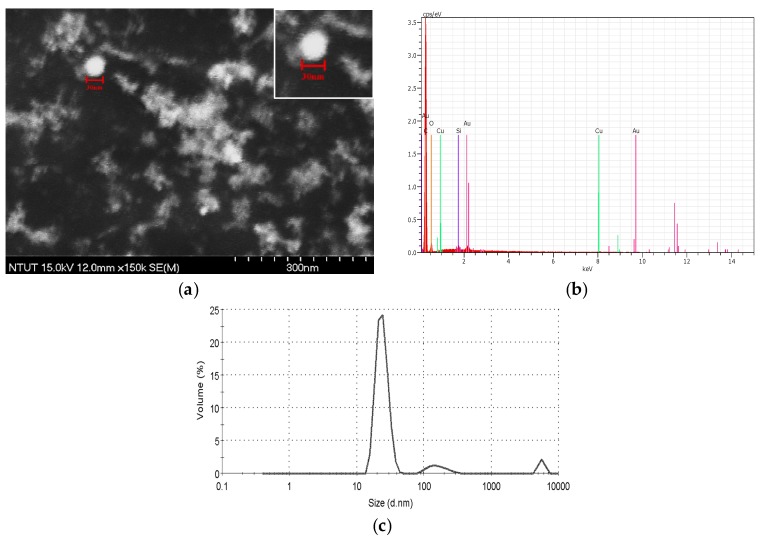
(**a**) Scanning electron microscopy (SEM) image; (**b**) energy dispersive X-ray (EDX) analyses of the elements of a nano-Au colloid; (**c**) size distribution in volume of nano-Au particles in the colloid.

Figure 3a shows a scanning electron microscopy (SEM) image of a nano-Au colloid fabricated through m-EDM. Each grid of the image is 30 nm; most nanoparticles are less than 30 nm in diameter, circular in shape, and agglomerated in several grids. Figure 3b presents the EDX results of the colloid, which indicate that the dominant element of nanoparticles fabricated through the Electric Spark Discharge Method (ESDM) is gold. This figure also shows different peaks, indicating that the colloid was not 100% pure and both carbon paste and copper gauze were used in the EDX analysis. Thus, minor elements (such as Cu, C, and O) identified in the colloid analysis are measuring errors. Figure 3c illustrates the size distribution of the nano-Au particles in the colloid. It indicates that most of the nano-Au particles fabricated by m-EDM are smaller than 100 nm. They are all nanoscale particles.

### 2.2. Suspension Analysis of Nano-Au Colloids Fabricated through m-EDM

**Figure 4:**
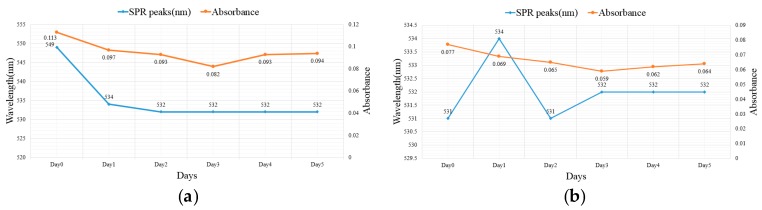

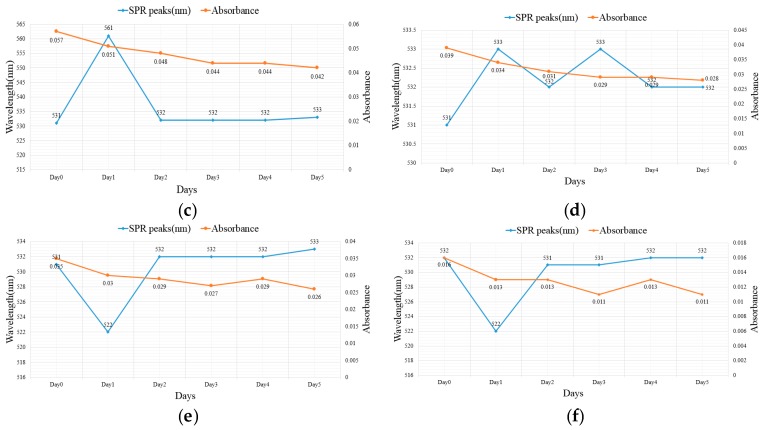
Results of an UV–Vis observation of the surface plasmon resonance (SPR) peaks and absorbance of nano-Au colloids fabricated at T_ON_–T_OFF_ settings of (**a**) 10–10 µs; (**b**) 20–20 µs; (**c**) 30–30 µs; (**d**) 40–40 µs; (**e**) 50–50 µs; (**f**) 100–100 µs.

Figure 4 compares the SPR peaks and absorbance from a 5-day UV–Vis observation of nano-Au colloids fabricated at different T_ON_–T_OFF_ settings. The square dots indicate the SPR peaks, whereas the circular dots denote absorbance. From the third day of observation across all T_ON_–T_OFF_ periods, the SPR peaks began to stabilize, whereas absorbance decreased consistently because some of the colloids continued to agglomerate and precipitate, causing their concentrations to decrease.

## 3. Discussion

In summary, this study used m-EDM to fabricate nano-Au colloids at different T_ON_–T_OFF_ settings with a total fabrication time of 1 min for each configuration. The SPR of the nano-Au colloid fabricated at the T_ON_–T_OFF_ of 10–10 µs was 549 nm on the first day of fabrication; it stabilized at 532 nm and exhibited a red shift (which indicated the shrinking of nano-Au particles) on the third day. In general, the peaks of nanoscale materials exhibit displacement, which is characterized by either a red or blue shift. A red shift means the wavelength is moving in the dominant direction and thus indicates that nanoparticles are agglomerating increasing in size. A blue shift indicates that nanoparticles are decreasing in size. Moreover, the absorbance of colloids varies depending on their concentrations: colloids with high concentrations, which are characterized by dense particle distributions, have high absorbance; whereas those with low concentrations have low absorbance. Accordingly, the nano-Au particles were either precipitated or dispersed in DW several days after they were prepared. Therefore, across all T_ON_–T_OFF_ settings, absorbance (i.e., concentration) correlated negatively with T_ON_–T_OFF_; absorbance peaked at 10–10 µs.

The SPRs of all nano-Au colloids stabilized at 532 nm, regardless of the T_ON_–T_OFF_ setting. The Zeta potential at 10–10 µs was −36.6 mV, suggesting high suspension stability in nano-Au colloids fabricated at this T_ON_–T_OFF_ setting; the nanoparticles of those colloids did not agglomerate. Table 2 presents a comprehensive comparison of all nano-Au colloids fabricated through m-EDM. SEM revealed that the particles in the nano-Au colloids were spherical in shape and less than 100 nm in size.

## 4. Materials and Methods

### 4.1. Fabricating Nano-Au Colloids through the Electric Spark Discharge Method (ESDM)

The proposed ESDM involves preparing nano-Au colloids in a simple, convenient, and low-cost manner, without the addition of any materials, and is an environmentally friendly nanophysical fabrication method for such colloids because, unlike costly vacuum EDM methods, it produces no waste [13].

**Figure 5:**
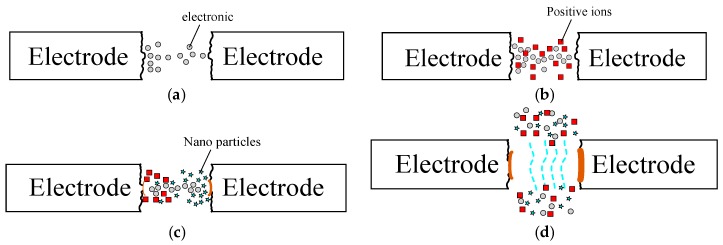
Illustration of the Electric Spark Discharge Method (ESDM). (**a**) Electrode surface surpasses the dielectric strength; (**b**) A discharge column appears; (**c**) The electrodes melt or even vaporize, ejecting their melted particles; (**d**) DW resumes insulation.

The ESDM delivers DC voltages of tens to hundreds of volts between two metal electrodes, and uses a servomechanism to control the cathode, allowing the electrode to inch toward the anode. Thus, when the distance between the two electrodes narrows to approximately 30 µm, the strength of the electric field located between two salient points on the electrode surface surpasses the dielectric strength of insulating liquids around the electrodes, leading to insulation breakdown in the liquids (Figure 5a). A discharge column appears between the electrodes and an electric discharge begins in the same position (Figure 5b) [14]. At a high arc temperature, the electrodes melt or even vaporize, ejecting their melted particles and allowing them to cool rapidly, freeze, and form nanoparticles in insulating liquid (Figure 5c). When the electric discharge ends, the discharge column disappears, eliminating nanoparticles, and DW resumes insulation (Figure 5d). This process is performed repeatedly to yield nano-Au colloids. In summary, the ESDM uses arc discharges to melt the surfaces of the anode and cathode, thereby yielding nano-Au colloids [15].

**Figure 6:**
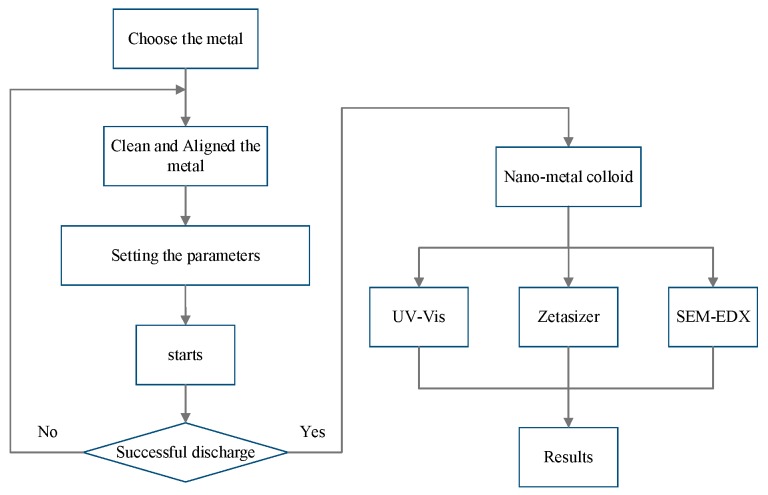
Procedure for fabricating nano-Au colloids through the ESDM.

Figure 6 depicts the procedure used for the fabrication of nano-Au colloids through the ESDM. First, two selected metal electrodes are rinsed with a proper volume of DW and attached in a beaker to connect to the apparatus. Second, after fabrication parameters (e.g., T_ON_–T_OFF_) are specified and the electric discharge time is adjusted according to the type of the metal, fabrication begins. Nano-Au colloids are obtained following the electric discharge. Finally, the colloids are examined through UV-Vis, a Zetasizer, and SEM–EDX [16].

### 4.2. Micro-Electrical Discharge Machine

**Figure 7:**
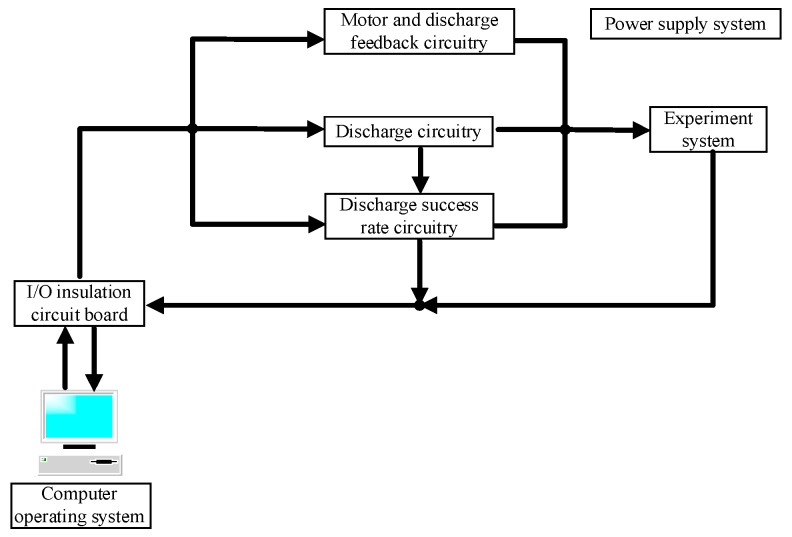
Configuration of m-EDM.

In this study, micro-electrical discharge machining (m-EDM) was used to ensure the continuity, reproducibility, and integrity of the processes and to optimize fabrication conditions. The duration of T_ON_–T_OFF_, the electricity consumption of the electrodes, the completion time, and the PID parameters can be specified in m-EDM, and the subsystems can be optimized [17]. An m-EDM apparatus is inexpensive, small, and easy to operate and maintain, and fabricates nano-Au colloids efficiently. The apparatus in this study comprised five subsystems: (1) computer operating system; (2) I/O insulation circuit board; (3) circuitry system; (4) power supply system; and (5) experimental system (Figure 7). Each of these systems is detailed in the following passages.

Computer operating system. The computer operating system uses VisSim to determine the start and finish time, PID parameters, and T_ON_–T_OFF_ of m-EDM. VisSim is a software program for the simulation and real-time control of dynamic systems. It efficiently and accurately executes various simulations—both linear and nonlinear, continuous and discrete, time-dependent and non-time-dependent. The program provides an integrated environment for system design. Its visual interface eliminates the need for manually written code. The VisSim environment in this study included a built-in RT/DAC4 board (a hybrid I/O platform with a personal computer interface (PCI) card) that used multiple digital and analog I/O modules to obtain data and control dynamic systems in real time. The RT/DAC4 board contained a field-programmable gate array chip responsible for computing digital signals.

I/O insulation circuit board. The name of the board was DB-8025. Because VisSim was installed on the host computer, the board separated the circuitry area from the host to prevent the host from being damaged by pulsed voltages or currents.

Circuitry system. This system comprised three subsystems: discharge circuitry, motor and discharge feedback circuitry, and discharge success rate circuitry. The details of these three items were as follows.

Discharge circuitry. This circuitry connected two 50 V DC power supplies in series to supply a voltage of 100 V and a current of 2.3 A. It used transistors to control the switch of the discharge loop. The discharge frequency of the circuitry was 0–50 kHz. T_ON_–T_OFF_ was specified through the computer. A light-emitting diode turned on when any electric discharge occurred and turned off when the discharge had finished. Pulse-width modulation (PWM) signals were supplied by the gates of IRF740 power field-effect transistors, which were in the conducting state at high potentials or in the cutoff state at low potentials. PWM signals were generated by VisSim; they were output by a motion control interface card and delivered through an optically coupled 6N137 guard circuit to the gates of the transistors.

**Figure 8:**
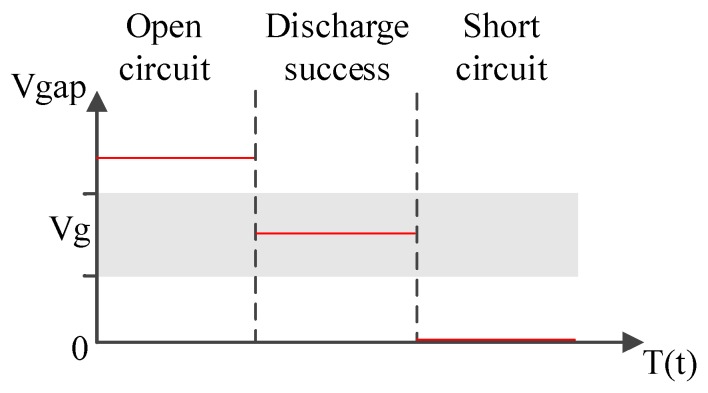
Adjusting the gap between the two electrodes in three different ways.

Motor and discharge feedback circuitry. This circuitry comprised a discharge loop circuit and motor circuit. The discharge loop circuit captured the voltage in the gap between two electrodes (Vgap), processed signals, and sent them back to the computer as feedback signals to control the motor. The signal of Vgap was delivered by a differential amplifier into a low-pass filter, thereby converting PWM signals into analog signals at a time constant of 1 ms (therefore, the analog signals had to be at least 10 times stronger than the cycle of discharge). The low-pass filter can be perceived as an integrator; thus, a PWM signal varied along with its analog level [18]. Analog signals converted from PWM signals were subsequently delivered through the I/O insulation circuit board to the computer. In the motor circuit, VisSim generated a voltage of −2.4 to 2.4 V to determine the sliding movement of the motor (i.e., going forward or backward) by the voltages Vg (which was within a specific range of voltage) and Vgap during discharge success. The gap between the two electrodes was adjusted in three different ways (Figure 8): (a) when both electrodes were in an open circuit and Vgap was at its highest level (higher than Vg), the gap between the electrodes became excessively wide and VisSim supplied a voltage of 0 to 2.4 V to the motor, allowing it to advance and slide forward; (b) when both electrodes discharged electricity successfully, Vgap decreased to the level of Vg; the gap between electrodes was 10–30 μm, and the motor remained motionless; and (c) when both electrodes were in short circuit and Vgap was zero, VisSim supplied a voltage of −2.4 to 0 V and the motor applied a force to slide the sliding table backward. This circuitry included a motor driver with a power-source voltage of 24 V and PWM as the output mode; the motor driver adjusted the PWM duty cycle in line with its voltage reference (REF+), which was directly proportional to the revolutions per minute of the motor. Electric-potential commands were delivered through an analog isolation circuit into the REF+ pin of the driver.

Discharge success rate circuitry. The differential amplifier captured Vgap and Igap as the output signals for a transistor-transistor logic circuit. The circuit determined the signals Vgap and Igap, which bypassed a digital isolation circuit and formed an AND gate with a 1 MHz pulse wave to output a pulse-wave signal to the counter pin. Afterwards, VisSim determined the number of successful electric discharges by the number of occurrences of the pulse wave and presented its estimates on a computer screen, which provided an overview of the electric discharges during nano-Au colloid fabrication.

Power supply system. This system comprised three circuit boards and a motor power supply.

**Figure 9:**
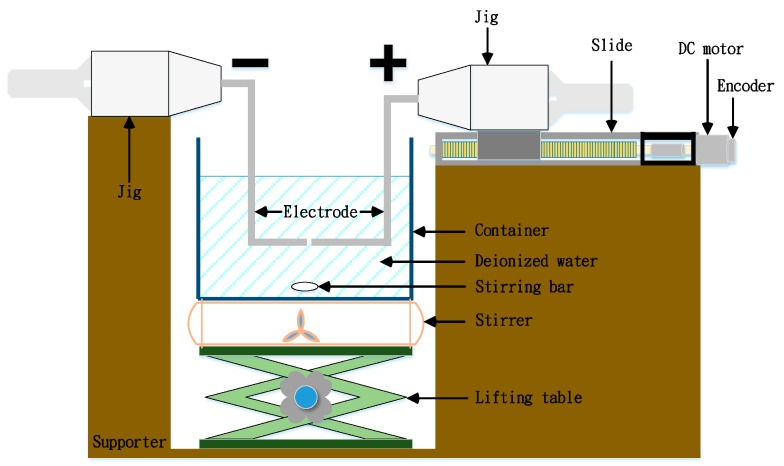
Structure of the electrodes of m-EDM.

Experiment system. This horizontally structured (Figure 9) system enabled easy operation. Vertical electrodes have curved shapes that present difficulties. The system in this study used horizontal electrodes because they were more useful than vertical ones. As Figure 9 shows, the left electrode was fixed by a jig to the support stand and the right was fixed to the sliding table. Because of its sheer size, the magnetic stirrer was replaced by a magnet-equipped fan that distributed nanoparticles evenly in the DW. A lifting table was placed on the lifting platform, which was raised during fabrication and lowered after fabrication. The support stand was designed exclusively for the lift platform. The two electrodes were attached to a pulse-wave power supply to discharge electricity and produce nano-Au particles, which were collected in the vessel. Powered by a DC motor, the right electrode moved up or down the sliding track (shown on the right side of Figure 9); the position of the electrode on the track was identified by an optical encoder.

### 4.3. Properties of Nano-Au Colloids

The absorbance and SPR of nano-Au colloids were determined through UV–Vis, Zeta potential and particle distribution through a Zetasizer (Malvern Zetasizer, Nano-ZS90, Worcestershire, UK), particle shapes and sizes through SEM (HITACHI, S-4700, Tokyo, Japan), and the constituent elements through X-ray (HITACHI, S-4700, Tokyo, Japan) [19].

The suspension stability of nano-Au colloids can be determined by their Zeta potential [20,21]. A high Zeta potential indicates an even distribution of nanoparticles in DW and high suspension stability, whereas a low Zeta potential suggests low suspension stability and causes nanoparticles to collide with each other and precipitate. In addition, because like electric charges repel each other, a Zeta potential with an absolute value of 30 mV indicates high suspension stability in nanoparticles [22]. 

## 5. Conclusions

In this study, the ESDM was employed with m-EDM to fabricate nano-Au colloids in DW at different T_ON_–T_OFF_ settings; the surfaces of two electrodes were melted by electric arcs, under normal conditions, and without the addition of any chemical substances. The contributions of this study are listed as follows.

ESDM can be used to fabricate nano-Au colloids without the addition of any chemical substances and under normal conditions. If applied in a medical settings, such colloids would cause no harm to the human body.

The UV–Vis, Zetasizer, and SEM–EDX analyses of the nano-Au colloids produced at different T_ON_–T_OFF_ settings (as determined through VisSim) confirmed the feasibility of fabricating these colloids and their nanoparticles through m-EDM using the ESDM. 

Nano-Au colloids fabricated at the T_ON_–T_OFF_ setting of 10–10 µs through the low-cost, easy-to-maintain, and self-designed m-EDM had a higher suspension stability and concentration than those of colloids fabricated with any other T_ON_–T_OFF_ setting. 

The wavelength of the SPRs of nano-Au colloids fabricated at different T_ON_–T_OFF_ settings stabilized at 532 nm, indicating no agglomeration or precipitation of nanoparticles.

m-EDM has the function of real-time monitoring, which can diagnose the problem when fabricating and improve the efficiency of m-EDM.

## Figures and Tables

**Table 1:** Parameters used for the fabrication of nano-Au colloids through micro-Electrical Discharge Machining (m-EDM).

**Temperature**	**Deionized Water**	**Electrode**	**Discharge Time**	**Voltage**	**Current**
25 °C	DW	Au	1 min	100 V	Approx. 6.5 A
**Atmospheric pressure**	**Volume of the beaker**	**Purity of the electrode**	**Diameter of the electrode (positive/negative)**	**On–off duration of pulse discharge (T_ON_–T_OFF_)**	**PID parameters**
1 atm	100 mL	99.99%	1/1 mm	10–10 µs	Kp = 0.21 Ki = 0.25 Kd = 0.015
				20–20 µs	
				30–30 µs	
				40–40 µs	
				50–50 µs	
				100–100 µs	

**Table 2:** Comprehensive comparison of all nano-Au colloids fabricated through m-EDM.

Days	Day 0		Day 5	
T_ON_–T_OFF_	SPR Peaks (nm)	Absorbance	SPR Peaks (nm)	Absorbance
10–10 µs	549	0.113	532	0.094
20–20 µs	531	0.077	532	0.064
30–30 µs	531	0.057	533	0.042
40–40 µs	531	0.039	532	0.028
50–50 µs	531	0.035	533	0.026
100–100 µs	532	0.016	532	0.011

## Supplementary Materials

Supplementary File 1
